# Remotely Piloted Aircraft Systems as a Rhinoceros Anti-Poaching Tool in Africa

**DOI:** 10.1371/journal.pone.0083873

**Published:** 2014-01-08

**Authors:** Margarita Mulero-Pázmány, Roel Stolper, L. D. van Essen, Juan J. Negro, Tyrell Sassen

**Affiliations:** 1 Department of Evolutionary Ecology, Doñana Biological Station, Consejo Superior de Investigaciones Científicas (CSIC), Seville, Spain; 2 Department of Sensor Science Technology, Council for Scientific and Industrial Research (CSIR), Pretoria, South Africa; 3 Centre for Wildlife Management, University of Pretoria, Pretoria, South Africa; University of Cape Town, South Africa

## Abstract

Over the last years there has been a massive increase in rhinoceros poaching incidents, with more than two individuals killed per day in South Africa in the first months of 2013. Immediate actions are needed to preserve current populations and the agents involved in their protection are demanding new technologies to increase their efficiency in the field. We assessed the use of remotely piloted aircraft systems (RPAS) to monitor for poaching activities. We performed 20 flights with 3 types of cameras: visual photo, HD video and thermal video, to test the ability of the systems to detect (a) rhinoceros, (b) people acting as poachers and (c) to do fence surveillance. The study area consisted of several large game farms in KwaZulu-Natal province, South Africa. The targets were better detected at the lowest altitudes, but to operate the plane safely and in a discreet way, altitudes between 100 and 180 m were the most convenient. Open areas facilitated target detection, while forest habitats complicated it. Detectability using visual cameras was higher at morning and midday, but the thermal camera provided the best images in the morning and at night. Considering not only the technical capabilities of the systems but also the poacherś *modus operandi* and the current control methods, we propose RPAS usage as a tool for surveillance of sensitive areas, for supporting field anti-poaching operations, as a deterrent tool for poachers and as a complementary method for rhinoceros ecology research. Here, we demonstrate that low cost RPAS can be useful for rhinoceros stakeholders for field control procedures. There are, however, important practical limitations that should be considered for their successful and realistic integration in the anti-poaching battle.

## Introduction

The two species of African rhinoceros, the black rhinoceros (*Diceros bicornis*) and the white rhinoceros (*Ceratotherium simum*) were driven to near extinction in the 1990’s [1]. Numbers of both species are raising in Africa since 2007 [2], but from 2010 the continued escalation in population growth has slowed down [3], and the two species are still vulnerable, with white rhinoceros classified as Near Threatened and black rhinoceros listed as Critically Endangered according to IUCN criteria [4].

South Africa holds more rhinoceros than any other country in the world, with 83% of Africa’s individuals, and also experiences the highest absolute levels of poaching, which is the main threat for their conservation [3]. Over the last years, and despite the anti-poaching efforts, there has been a massive increase in the number of rhinoceros poaching incidents. In 2010 there was an average of 0.9 rhinoceros killed per day; in 2011 it increased to 1.2; this number escalated to 1.8 in 2012, (resulting in 668 deaths along the year) and it has reached a staggering historical record of 2.2 per day in the two first months of 2013 (up to February 20^th^) [3].

The rhinoceros poaching is a complex problem with multiple causes and potential solutions [5]. Their horn is considered to be a traditional medicine for a variety of ailments in Asia [6], with the highest demands from China, Hong Kong, South Korea and Southeast Asian countries, and it is used for ceremonial purposes in Yemen [7], [8]. Due to the high demand and the illegal nature of the trade, the prices fetched by the horn in the black market are high. This constitutes a temptation to rural people with scarce resources, as the market value of one horn-set may be equal to the salary of several years for the poacher [5].

There are various long and medium-term strategies in progress to reduce the illegal trade of rhinoceros horn, and they remain in constant discussion: horn control, legislation, cooperation with the horn purchasing countries, environmental education and rural development projects in rhinoceros areas, most of them conducted by public institutions or NGOs [2], [7]. These general strategies are also supported by immediate anti-poaching actions in the field, directed by the management authorities or the landowners, and carried on by either park rangers or security companies.

In South Africa, around a quarter of the total population of rhinoceros live on private land [2]. The owners of these reserves and game farms are increasingly hiring specialized companies that focus on the protection of wildlife and the apprehension of poachers. The service of protecting valuable wildlife has led to an emergence of this type of business in recent years. They employ techniques based on operational methods of the police and armed forces. The basis of this strategy is to deploy ground based patrol units that spend multiple days tracking animals and poachers, and monitoring the fence lines for breaks. While the cost of employing these companies is high (around 10,800 € per year to maintain 1 guard patrolling 700–800 ha), they are the most popular alternative to reduce the number of poaching incidents in private land. Both private companies and public agents working in rhinoceros anti-poaching are demanding new technologies to increase their efficiency to detect and intercept poachers before a rhinoceros is killed. The need to be more effective in addressing the poaching problem was expressed by the IUCN/SSC African Rhinoceros Specialist Group [2].

Discussions with security companies and conservation agencies have indicated that aerial monitoring may be of assistance in covering more ground, and remotely piloted aircraft systems (RPAS hereinafter) have been suggested to do this work [5]. Some security firms already patrol the vast farms by flying twice a day with a micro light aircraft and directing the “boots on the ground” to the whereabouts of the rhinoceros.

Remotely Piloted Aircraft Systems (RPAS), sometimes also referred as Unmanned Aerial Vehicles (UAVs), Unmanned Aerial Systems (UASs) or drones (the ones for military purposes), are aircrafts (fixed or rotary wings) that are equipped with cameras and/or other sensors and can be sent (using manual, semi-automatic or automatic control) to a destination to gather information. These aircrafts act like an “eye in the sky” [9] with the operator at the ground control station receiving data or sending orders to the aerial platform. RPAS have been used for locating “enemies” in military applications for the last 20 years [10], and more recently they have started to play a role in many civilian tasks, including wildlife monitoring [9], [11]–[15].

In this paper, we describe the use of a small low cost RPAS equipped with three different types of cameras to test their ability to support rhinoceros anti-poaching tasks in cooperation with a specialized security company working in the KwaZulu-Natal province of South Africa. We performed several flights in order to test the technical capabilities of the system to detect rhinoceros, to reveal simulated poachers and to do fence surveillance. We evaluated the effectiveness of the system at different altitudes and times of the day and night, and over the two main habitat types in the area: open grassland and forest. Considering the most common *modus operandi* of poachers, we analyzed the aspects that affect remotely piloted aircraft’s integration in anti-poaching operations.

## Materials and Methods

### Ethics statement

At present, no regulations are in place for the use of RPAS in South Africa. Draft regulations pertaining to the use of UAVs have been published by the South African Civil Aviation Authority (SACAA) but these have not been ratified to date. The Recreational Aviation Authority of South Africa (RAASA) indicated that the flights could be performed as long as they were conducted over wildlife areas with low manned aircraft activity and not close to registered active airfields. The study therefore complies with the current South African legislation involving aviation safety. The RPAS operators had the required international radio operator licenses to operate in the frequencies used for this work.

To get an insight into the poaching problem, we met four people involved in rhinoceros protection at different levels. These interviews did not contain personal or ethically sensitive information, therefore ethics approval was deemed unnecessary by both the Ethics Committee of Animal Welfare of Doñana Biological Station (CEBA-EBD) and the Animal Ethics Committee (AEC - Faculty of Natural and Agricultural Sciences), a sub-committee of the Committee for Research Ethics and Integrity of the University of Pretoria. All four interviewed people provided their verbal informed consent to take part in the study once informed about the nature and objectives of the investigation. The participants gave their implied consent through cooperation and it was therefore deemed unnecessary to obtain written consent. All aspects of these personal communications were written down as part of the data collection process of the entire project. Ethics committee approval was deemed unnecessary to approve this consent procedure. We thank farm owners and the security company for providing valuable information used in this study, the lodging and the logistics for the field campaign.

### Study area

The study area comprised 13 farms whose areas ranged between 1,500 and 25,000 ha, covering a total of 100,000 ha located in KwaZulu-Natal province, South Africa. The habitat on the farms is a combination of forest patches and grassland, and is utilized mainly for ecotourism and hunting. The rhinoceros population (both black and white) in the area is approximately 500 individuals. The field campaign was performed during August 2012.

### Rhinoceros safety requirements definition

To define poachers’ way of operation and actual anti-poaching surveillance methods, we separately met four people at the onset of the fieldwork: the security company manager, the rangers’ coordinator and two rangers of the farms of the study area, all of them responsible for different aspects of rhinoceros safety.

#### Airframe

**Figure 1:**
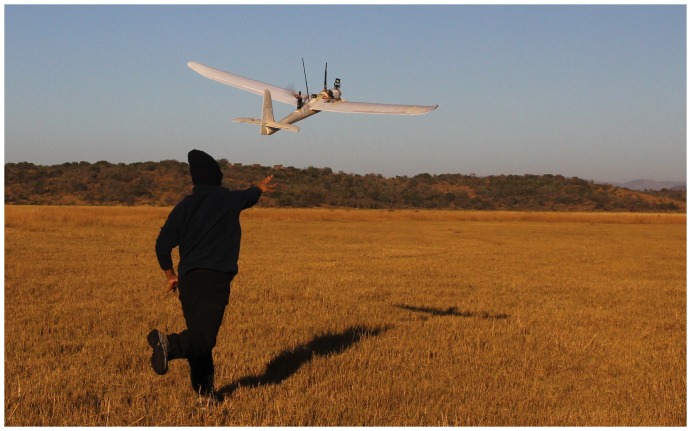
Remotely Piloted Aircraft taking off.

The fixed-wing RPAS is a commercially available radio control plane airframe Easy Fly St-330 (St-models, China) modified by our team. It has a wingspan of 1,960 mm and a maximum take-off weight of 2 kg with a 350 g payload (Fig. 1). It has a maximum range of 10 km; an endurance of 50 minutes and it is launched by hand and landed manually in small patches of open terrain. It is propelled by a brushless electrical motor using a lithium polymer battery.

The plane is capable to operate in three different modes, and it is possible to switch from one to the next during the flight: automatic (using the abilities of the autopilot), FPV (“first person view mode”) and manually (radio control conventional mode, also called “third person mode”). It is equipped with an onboard FPV video camera, a GPS (10 Hz, Mediatek, model FGPMMOPA6B), a data-logger with a barometric altitude sensor Eagletree GPS logger V.4 (Eagletree systems, WA, USA) and an autopilot (Ikarus, Electronica RC, Spain) which provides flight stabilization and On Screen Display (OSD). The OSD provides GPS information about the position, speed, height and course of the aircraft. The data combined with the FPV video signal from the camera are sent to the ground station. For nocturnal flights we equipped the plane with a set of LED lights of different colors in the wings, nose and tail that allowed the pilot to locate and position the aircraft visually.

#### Ground control station

The ground station contains a monitor, a DVD recorder, a video receiver and a control signal transmitter with its associated antennas. It also includes a Laptop PC to program the autopilot, store the pictures and data logs, and decode in-flight telemetry, allowing tracking the position of the RPAS in real time on a Microsoft map (Redmond, WA, USA).

#### Payload

Due to the RPAS payload limitations, only one of the cameras can be utilized on each flight.

Still photo camera: Panasonic Lumix LX-3 digital photo camera 11 MP (Osaka, Japan). It is integrated in the plane wing and aimed vertically to the ground. The camera is activated during the flight at the desired point using a mechanical servo. It is set in speed priority mode and in its widest zoom position.

High Definition (HD) Video Camera: GoPro Hero2 (Woodman Labs, Ca., USA). It has a field of vision of 127° and a resolution of 1080 p (1920×1080). The video camera is integrated in the nose of plane aimed forward and downwards, at an angle of 30° below the horizontal.

Long wave uncooled thermal video camera: the infrared camera module is a Thermoteknix Micro CAM microbolometer with a resolution of 640×480 pixels. The lenses of the module are interchangeable and tests were done with a focal length of 18.8 mm and 1.2 maximum aperture lens. This equates to a diagonal field of vision of 39.8° respectively. This camera can be integrated in the plane wing aimed to the ground at 15° nadir or in the same position but with at an angle of 30° below horizontal. Price of all the RPAS components is shown in table 1.

**Table 1:** Cost of the RPAS equipment (Material bought in Spain in June 2012).

Component	Price (€)
Airframe with the electronic system	1,000
Ground control station (antennas included)	6,000
Stills Photo Camera	450
HD Video camera	300
Thermal camera	6,000
Total	13,750

### Experimental procedures

We conducted a total of 20 flights. On each flight, we passed over the targets at altitudes ranging from 10 to 260 m above ground level (AGL). Flight speed varied due to wind speed and direction, with a minimum of 15 km/h on the windiest days flying against the wind, up to 50 km/h when flying with tailwinds. In eight of the flights we mounted a still photo camera, eleven flights incorporated a thermal video camera, and only one incorporated a HD visual video camera. Four of those flights, with the thermal camera, were conducted at night, and the rest of them were performed during daylight.

Rhinoceros detection flights were done over approximate rhinoceros locations previously provided by rangers monitoring individuals regularly on the ground. Poacher detection flights were performed over areas where rangers and members of our team dispersed simulating poacher activity. We flew along the fences in first person view mode, which means using the real time video transmitted from the RPAS to the ground station, and the pilot guiding the plane manually using the transmitter.

### Data analysis

Pictures obtained with the Panasonic LX3 camera were reviewed to identify rhinoceros, people or fences. They were geo-referenced using the information provided by the onboard Eagletree GPS logger V.4 (Eagletree systems, WA, USA) that includes a barometric altitude sensor. The software for geo-referencing is a customized extension that we developed with ENVI (Exelis Visual Information Solutions, CO, USA) that combines our plane position data with the pictures to generate GeoTIFF files. We projected the geo-referenced images using ArcGIS v.10 (ESRI, Redlands, CA, USA) to check that the whole desired area was actually covered.

The time invested in photo reviewing was 3.5 seconds per picture on average. To process each plane track took us 15 minutes and the geo-reference process was around 3 seconds per picture. One observer was able to do all the processing simultaneously, as he could first process the track, then start the geo-referencing program to run and do the review of the pictures while the geo-reference program was working. On average, an observer with a computer needed around 45 minutes to process a 500 pictures flight, which is the usual number of pictures taken per flight.

Overlapping the images obtained depends on flight altitude and plane speed, and was calculated according to the equation:
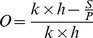



Where:

*O* is overlapping (%),

*h* is altitude AGL (m),

*S* is speed of the plane (m/s),

*P* is the number of pictures the camera takes per second. *P  =  2* in our camera,

*k* is a constant that depends on camerás vertical sensor dimension. The equation to calculate it is:
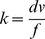



Where:

*dv* is vertical dimension of the sensor (5.6 mm in our camera)

*f* is local length (5.1 mm in our camera)


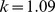
for the camera we used.

Spatial resolution of imagery depends on the altitude at which images are taken and the camera sensoŕs characteristics. With the camera we used, the relationship between altitude AGL and resolution was as indicated by Rodriguez *et al*. (2012).
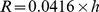



Where *R* is Resolution (cm),

*h* is altitude AGL (m).

The area covered by the pictures can be calculated considering the flight altitude, the speed of the plane and horizontal dimension of the camera sensor. 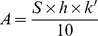



Where *A* is area covered by the plane/time (ha/h),

*S* is speed of the plane (km/h),

*h* is altitude AGL (m),

*k’* is a constant that depends on camera horizontal sensor dimension. The equation to calculate it is:
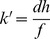



Where *dh* is horizontal dimension of the camera sensor (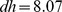
mm in our camera).

*f* is local length (5.1 mm in our camera)


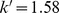
for the camera we used.

Deviations from the horizontal plane, mainly produced by wind, caused some distortion in some of the pictures, but it did not affect our objectives. HD and thermal camera videos were reviewed to identify targets: rhinoceros, people or fences. We extracted video frames using Adobe Premiere Pro CS5 and improved their image quality using Adobe Photoshop CS5 (Adobe, CA, USA). Due to the forward and downward angle of the video cameras, it is not possible to project the video frames horizontally on the map, but by contrasting the time corresponding to the frame with the plane track file, it was possible to place the targets with a 50 m precision.

### Images analysis

We selected the pictures and extracted the video frames that contained targets. Many of them appear in consecutive pictures due to overlapping. To establish a reference altitude each time a target was detected, we chose the image in which the target appeared more centered on the picture area. If a target was overflown more than once in the same flight but in several turns, the different detections were considered, as the observers who analyzed the images did not know the plane trajectory or the target locations, so they did not know if the targets where the same or different. If two targets were detected on the same picture, we classified them separately because the quality for each one can be different. Images were classified according to their quality following these criteria:

-High: the targets are detected and identified at first glance of the picture or video. Fence poles and wires are visible.

-Medium: the target is detected on a second or third review of the picture or video. To identify the target, it is necessary to zoom in, check other consecutive pictures, review the video in slow motion, or post process the picture or frame (modify the contrast or increase brightness). Fence poles are visible but wires are not distinguishable.

-Low: an object is detected but its identification is not possible. Fence trajectory is detectable but the poles or wires are not distinguishable.

We assessed the detection of the targets on each flight considering that they can be: 1) confirmed: when the target is identified with high or medium quality images and 2) not confirmed: when the target identification is not possible, either because the target could not be found at all or because the images had a resolution precluding identification.

Habitat type was characterized according to vegetation coverage in 100 m around each target location as: 1) Forest: vegetation coverage > 75%, 2) Grassland: vegetation coverage <75% and 3) Mixed: refers to the cases where the targets are located at the border between two farms. These locations have fences with maintenance trails along, so even presenting a high percentage of vegetation cover around, they could still be considered as open areas from a detectability perspective.

To facilitate the evaluation of the detectability according to time of day, we divided the flights in four periods related to light conditions: morning (07:00–10:15 h), midday (10:16–14:00 h), evening (14:01–17:45 h) and early night (17:46–20:00 h). Times are in South African local time. As a reference, in the study area, sunrise was from 6:31 h to 6:59 h and sunset from 17:44 h to 18:00 h, from August 1^st^ to August 31^st^.

## Results

### Poachers’ modus operandi, poaching surveillance and rhinoceros monitoring (field interviews)

The people we interviewed provided very similar comments about their perception of poaching activities. This was not surprising as all of them work in the same area and deal with the same problem, although it is noticeable that the people at different work levels are able to provide detailed information about the whole poaching topic (from a general perspective to specific field issues), evidencing that there is a good information flow among rhino protectors.

The most common profile of a poacher is that of local people with low income and who obtain money selling the rhinoceros horns to the lowest levels of the syndicates. The poacher accesses the game farm on foot, sometimes accompanied by dogs, and generally there is an accomplice who drives him close to the fence and meets him at some point for collecting. Poacher entry hot spots onto the farms are generally through the same areas: near roads, trails, villages or known rhinoceros territories. The poacher enters the game farm either by cutting a hole in the fence, climbing over it, or crawling underneath it.

Poachers do not show preferences for particular times of the year, days of the week or time of the day, although there are some variations according to the season. Considering nights only, they show a preference for full moon nights (rather than dark nights) to enter the game farms, as increased lightness facilitates their movements. In summer there is more water available, and consequently the rhinoceros and the poachers are more dispersed, which makes it more difficult to detect them. In winter the rhinoceros gather near waterholes, therefore the poachers concentrate on the areas with available water and there is also less vegetation for camouflage. Time poachers spend inside the farm typically ranges from 3 hours up to two days.

The most common method for killing the rhinoceros is by shooting them with homemade or cheap firearms. Poison is also used in the form of anesthetic injected into apples or other fresh fruits that poachers leave close to waterholes used by rhinoceros. Snaring with thick wire or cable snares are also used but not on a regular basis.

Current monitoring of rhinoceros is generally based in aerial surveys (once per year) combined with GPS data of the animals provided weekly by field teams. Surveillance of farm perimeter is generally done every two days, or daily if there are poaching alert signals. Farm neighbor’s cooperation on anti-poaching is generally well established, especially if they use the services of the same security company.

General surveillance procedure in our study area consists on 90 guards patrolling the 100,000 ha on a daily basis. Standard cost of poaching control including vehicles, fuel, materials and the rangers’ salary, is about 900–1,000 €/ 700 ha/ month. An additional cost related to poaching is fence maintenance, done either by the landowner or by the security company. Fence maintenance cost can vary substantially from year to year and is not only associated with poaching but also with animal damage or natural deterioration. Other anti-poaching actions in which landowners and security companies are involved in include cooperation with wildlife surveillance teams and participation in environmental projects with local communities.

### Flight data

We present a description of the results of the 20 flights and the scenarios where the targets were located in Table 2. No alarm reaction or flight responses were detected from any animals caused by the plane in any of the RPAS flights.

**Table 2:** Flights results.

Camera	Time period	Time start	Time end	Target	Habitat	Result	Altitude (m) (Min-Max)
Still photo	Morning	09:03	09:26	People	Grassland, Mixed	Confirmed	32–149
				Fences	Mixed	Confirmed	40–175
		09:05	09:38	Rhinoceros	Forest	Confirmed	57
		09:42	10:02	People	Mixed, Forest, grassland	Confirmed	29–82
				Fences	Mixed	Confirmed	42–72
		09:52	10:12	Rhinoceros	Forest	Confirmed	31–137
	Midday	10:16	10:39	Fence	Mixed	Confirmed	50–175
				People	Grassland	Confirmed	123–158
		11:22	11:43	Rhinoceros	Grassland, Forest	Confirmed	38–239
		13:14	13:56	People	Forest	Not confirmed	
	Evening	17:19	17:38	Rhinoceros	Forest	Confirmed	82
				People	Grassland, Forest	Not confirmed	
				Fences	Mixed	Not confirmed	
Thermal video	Morning	07:51	08:11	Fence	Mixed, Grassland	Confirmed	27–155
				People	Mixed, Grassland	Confirmed	31–100
		08:21	08:55	Fence	Mixed	Confirmed	37–98
				People	Mixed	Not confirmed	
		08:27	08:56	Fence	Mixed	Not confirmed	
		09:25	10:03	Fence	Mixed	Confirmed	48–54
				People	Mixed	Not confirmed	
	Midday	10:27	10:46	Rhinoceros	Forest	Not confirmed	
		10:40	11:07	Rhinoceros	Forest	Not confirmed	
		12:32	13:04	Rhinoceros	Forest, Grassland	Not confirmed	
	Night	18:19	19:02	People	Grassland, Forest	Confirmed	12–125
				Fences	Mixed	Not confirmed	
		18:41	19:00	Rhinoceros	Forest	Not confirmed	
		19:17	19:40	Fence	Mixed	Not confirmed	
				People	Grassland	Confirmed	36
		19:27	19:45	Rhinoceros	Grassland	Not confirmed	
Visual video	Midday	11:08	11:27	Fences	Mixed, Forest, Grassland	Confirmed	10–17
				People	Mixed, Forest, Grassland	Confirmed	10–35

We provide the minimal and maximum altitude at which a target was confirmed in each flight. When only one value is presented it means that the target was located just once.

### Still photo camera data

The pictures covered the area overflown by the plane with an overlapping between 36.3% in the flights at highest speed and lower altitudes (10 m AGL and 50 km/h) and 99.2% at lowest speed and highest altitude (260 m AGL and 15 km/h). As an example, flying during one hour, at an altitude of 150 m and a speed of 30 km/h we were able to cover 711 ha. Resolution varied from 0.4 cm in the pictures obtained at the lowest altitude to 11.8 cm resolution at the highest.

**Figure 2:**
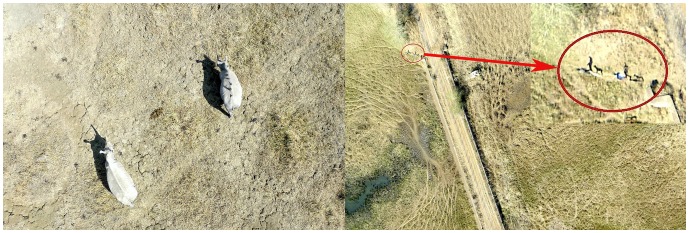
Images obtained with still photo camera. Left: Two rhinoceros (altitude 44 m AGL) in grassland habitat. Right: two people accompanied by two dogs near the fence (altitude 123 m AGL). These images were classified as “high quality”.

Rhinoceros were easily detected in both grassland and forest habitats at a minimal altitude of 31 m and a maximum of 239 m AGL (Fig. 2). People simulating poachers were identified in a wide range of altitudes from 29 to 158 m in grassland and forest habitat, although it was more difficult to distinguish some individuals in the forest, especially certain rangers in camouflage clothing because they offered less contrast with the surroundings. Fence surveillance results were acceptable at morning and midday hours, with the pictures presenting enough quality to zoom in and find people along it. At the lowest altitude (40 m) it was also possible to detect footprints in the sand, but the quality was not sufficient to check the condition of the fence wires along the entire fence route. (Fig. 2)

The quality of the images was best at midday (80% of the pictures had high quality in this time period) with vertical sunlight, and the results were worse when the shades of the trees produced dark areas, which happened in the morning (66% high quality) and in the evening, when this effect is accentuated because the air is less clean causing a blurry effect (100% medium quality pictures).

### Video data

**Figure 3:**
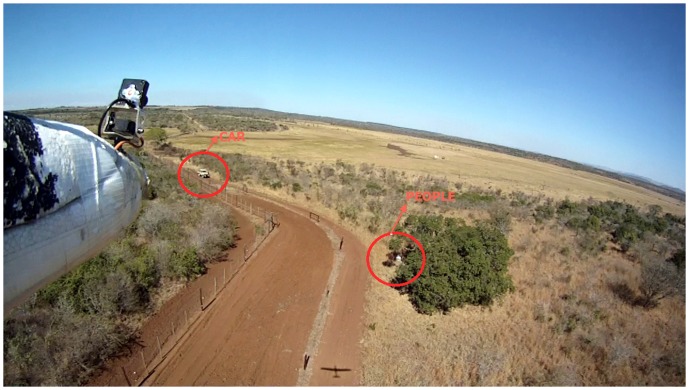
Frame extracted from HD video. People and car near the fence. This image was classified as “high quality”.

The HD video camera provided good resolution below 40 m AGL, but due to the wide angle of the lens (fov 127°), flights above 50 m altitude AGL had not enough quality to identify people or to survey the fences. These results led us to cancel the planned flights for rhinoceros detection, as we considered the altitude had to be so low to identify objects that it could be dangerous for operating the airplane and might also disturb the rhinoceros. (Fig. 3 and Video S1)

**Figure 4:**
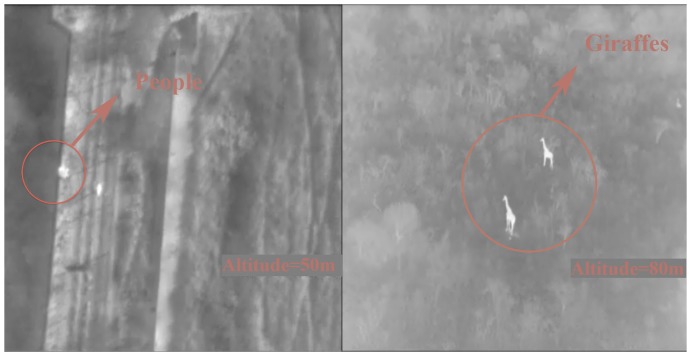
Frames extracted from thermal video camera. Left: A person near the fence (medium quality image). Right: two giraffes captured during one of the flights. Although giraffes were not the targets of our study, this image may serve as an example of the quality of thermal captures when thermal contrast is high.

The thermal camera provided the finest images in the early morning, when the ground was coldest and there is more contrast between it and any animal or person. We confirmed the presence of targets at altitudes as high as 155 m, but in general, it was difficult to identify them at the species level, as they appear in the video as diffuse (although very contrasting) white spots. Only 5% of the images taken with this camera presented high quality, 24% medium and 71% low. At the earliest hours of the night, the results obtained did not allow us to confirm that any of the spots we detected when overflying a rhinoceros was actually a rhinoceros, and low altitude was needed to identify the people using details such as body shapes. After hours of working with thermal video and “training the eyes” we noticed a considerable improvement on detection and shapes identification. Resolution offered by the thermal camera was enough to follow fence posts and to detect individuals, but fence wires were not distinguishable at all. (Fig. 4 and Video S2)

## Discussion

Rhinoceros poaching is a pressing issue that needs immediate solutions in the field. Rhinoceros stakeholders are demanding new technologies [2]; social media have already suggested the use of drones [16] and WWF announced in 2012 that it sponsors an on-going remotely aerial survey system and anti-poaching program in cooperation with Google to protect tigers, rhinoceros and elephants [17]. RPAS have already proved their efficacy for military and civil applications in general, and wildlife monitoring in particular. Now the question is how to integrate RPAS in rhinoceros anti-poaching tasks. To answer this question there are two main aspects to consider: capabilities (technical and practical) and current limitations.

### Technical considerations

The still photo camera provided the best results in terms of image quality (94% of the pictures taken by this camera allowed us to confirm the targets) and precision in the location. That is why this is the most attractive and currently the method of choice in conservation biology studies [18], [19]. However, it is a relatively slow procedure, as images must be downloaded after RPAS lands and then reviewed and post processed. Even if pictures were transmitted in real time to the ground station (which is technically possible) accelerating the process, it would still take time to review them. Therefore, the use of a still photo camera would not be suitable to support real time anti-poaching tasks like poachers location during a pursuit. A positive aspect is that still photos would be the best method to provide image proofs against poachers because it offers the best resolution.

Video offers real time data, so it seems a better option than still images for poaching control. It is recommended to use a video camera with a narrower view field and zoom capabilities to identify the targets at safe altitudes (over 100 m AGL) in real time with enough magnification. Although video offers less precision on target location, according to the interviews with the people involved in rhinoceros safety, accuracy is not so important for anti-poaching purposes, or at least it is less important than immediacy.

As far as we know, this study offers the first nocturnal tests for wildlife monitoring using thermal cameras onboard a fixed-wing small RPAS, which is the only option for RPAS nighttime surveillance. The camera we used provided acceptable results when flying low, but the quality does not guarantee to identify some targets and it is possible to miss some, even one as conspicuous as a rhinoceros, when thermal contrast is low or flying at high altitudes. 29% of the thermal images allowed us to confirm the targets, and the rest presented low quality, precluding identification. It is important to consider that the last are still useful, as in a real anti-poaching situations, the dubious objects could be further inspected either overflying lower the RPAS or by other means (as ground patrols). Additionally, the quality and resolution of the thermal sensor can be improved and therefore the detection.

As expected, habitat type had an influence on target detection, which is more noticeable when using visual cameras, either video or still photo. Although rhinoceros are large enough to be detected from high altitudes with still photo cameras, people, especially if wearing camouflage clothes or hidden under a thick tree may not be detectable if flying at high altitudes.

Time of day had an influence on target detection. Our results indicated that best time for the use of visual cameras was from early morning to midday, and decreased along the evening. Thermal camera provided better results when temperature contrast is higher [20], mainly at early morning and night. The detectability limitation linked to the hourly cycle, which is related to light conditions and air-ground thermal contrast, is important, as this means that the usefulness of RPAS as monitoring tools does not remain constant throughout the day. This effect would be accentuated when the temperatures are higher and humidity increases, as we would expect in the area where we performed the tests during summer, or in places with high humidity levels (tropical or coastal areas).

There is a compromise in deciding flight altitude for anti-poaching. Lowest altitudes provide the best results in terms of image or video resolution, but the surveyed area is smaller. Flying low implies more risk for the plane in case of failure and easier detection of the plane from the ground (therefore disturbing the rhinoceros or being more easily detected by poachers). Our results suggest that an altitude range between 100 and 180 m AGL is suitable for detecting rhinoceros or people, and to do fence surveillance with acceptable quality levels, it is a safe altitude for the plane and it is not very noticeable from the ground.

### Practical considerations

Considering poachers *modus operandi* and current security procedures, there are some limitations for the integration of RPAS in routine anti-poaching work in a realistic and efficient manner.

#### Legal aspects

South Africa, as with many other countries in the world, does not yet have a legal framework for operating unmanned aerial systems. The absence of regulation for flying beyond line of sight constrains the range of work of the aircrafts, strongly limiting the actual technological capacities of the systems to just short range operations of RPAS operated by manual radio control [21], as the ones we presented in this paper. Some authors already addressed this issue arguing that operations that do not pose a safety threat to humans in the air or on the ground should be permitted [22]. They suggested Light UAVs for poaching site surveillance and proposed ideas including UAV corridors, avoiding inhabited areas and frequently used airspace, all in order to fly these aircrafts safely. We support these proposals, as rhinoceros distribution coincides with very low populated areas where the risk of hitting a person or crashing with another aircraft or infrastructure is low, especially flying at altitudes below 300 m AGL. The South African Civil Aviation Authority (SACAA) has published draft UAS regulations [23], [24] that include exceptional permits for public interest uses of UASs (as anti-poaching could be classified). However, to date there has been no official notice that the SACAA has approved any protocol for UASs flights.

#### Scale of work and range

Scale of work is a limiting factor in using RPAS for anti-poaching tasks. The territories rhinoceros inhabit are large and population density is low (1 rhinoceros/200 ha on average in our study area). We demonstrated that it is possible to have an “eye in the sky”, but this eye cannot look everywhere all the time, so that logistics have to be evaluated. How many eyes are necessary and how often do they have to look? The management and application of a RPAS or multiple RPAS is a key question that rhinoceros safety stakeholders need to consider and define before planning RPAS use.

Small low cost RPAS typically fly for 30–40 min and their range is limited up to 10–15 km. Roughly considering that a RPAS flying at 150 m AGL could cover 711 ha, to survey the 100,000 ha of our study area would take around 140 hours (5.8 days). And that excludes the time to move the Ground Control Station from one point to another, taking off and landing, changing and charging the batteries, data processing, and assuming 24 hours personnel availability. Obviously, that time would be reduced if having more RPAS available, but that would entail higher associated costs.

There is a compromise between the area to control and the frequency of this control. A reasonable solution would be to focus RPAS for monitoring hot spots: either rhinoceros preferred locations or most sensitive poaching areas, which are generally known by security companies or park rangers, or areas where access by anti-poaching patrols and/or vehicles is complicated by other factors such as difficult terrains etc.

#### Weather conditions

Small RPAS are safe to fly up to 15–20 km/h wind speed. They are not suitable to operate in rainy conditions because the electronics can be damaged and the data obtained by the cameras in low light levels would not be useful.

Temperature and terrain altitude affect air density, which influences the power needed to fly the plane, aircraft battery consumption and consequently endurance and range. These variables also influence the power required for takeoff, which is higher the colder it is, or in higher terrains. This can also translate into more failed takeoffs. In experiments performed for other purposes, we found that our system lost 10 minutes of endurance (around 30%), when comparing sea level in summer in Spain to winter at 2,000 m in South Africa.

#### RPAS possible negative effects

Rhinoceros did not show any alarm or discomfort reactions during our flights. However, there is no proof that RPAS could not disturb them or other animals if their use is continuous, so further investigation of this aspect is needed. Some farms that have rhinoceros also offer ecotourism activities that bring important income. Therefore, visitor acceptance to the presence of RPAS in those areas would be important.

#### Choosing the right RPAS

The range of RPAS available is extensive and growing by the day. From micro systems that fit in the palm of a hand up to 2 tons airplanes, there is a huge variety in market offer. Considering the scale of work, the funding limitations and the sensor requirements, “close range” [25] RPAS seem to be the best choice for anti-poaching purposes.

RPAS’ users always want to improve system performances to maximize endurance, range and sensors capabilities (data quality), and to minimize another set of characteristics associated with the RPAS: price (of the system and spares), logistics (size, transportation, taking off and landing requirements), and experience level needed for its operation. Unfortunately, any improvement in the system performances entails an undesirable effect in one or more of the second set of characteristics that would make RPAS less affordable or practical. Thus, the most suitable choice is a balanced compromise the user has to accept considering all the pros and cons for his specific purposes.

#### Costs and benefits

The recommended close range RPAS are typically lighter than 5 kg, have 30–45 minutes endurance and offer an operational range between 5–20 km. The price, capacities and reliability vary according to the manufacturer. In general, there is an investment in a whole system, composed by the ground control station, antennas, and two or three planes that need to be repaired or substituted when they reach a certain number of flights. As a reference, the system we used has performed more than 500 flights with an approximate total investment of 14,000 € including the sensors payload (see Table 1). There are more affordable options available in the market, but from our experience, reliability of some very cheap components like servos, batteries or even tripods is not guaranteed and their failure may cause serious problems affecting expensive components, so it is worth to get at least medium quality spares.

The benefit of integrating RPAS in anti-poaching work is difficult to evaluate in economic terms, as its calculation would involve to put a price on the life of a rhinoceros and to evaluate how many could be saved by using RPAS. It has been pointed out [26] that white rhinoceros carry two types of values: a commercial value (live rhinoceros trade and rhinoceros hunting) and a conservationist or aesthetic value. The first one could be calculated (white rhinoceros average price in 2012 was 17,330 €, record price in 2012 was 53,784 €; black rhinoceros record price in 2012 was 44,969 €) but the second one is hardly translated into numbers. Currently there is not real work using RPAS to be able to estimate the number of rhinoceros that could be saved by RPAS use or to calculate other types of surveillance costs that might be reduced by using this technology. As a reference, the investment needed for a small low cost RPAS (including spare platforms, spares, tools, etc.) that could last for about two years being used weekly (around 30,000 €), plus around 6,000 € to train operators, could be assumed by a medium size security company or institutions that control areas between 50,000–100,000 ha (Security company manager, pers. comm.). The business of anti-poaching is growing, especially in private land, with the result that RPAS will be not only appreciated for their real usefulness, but also as a competitive asset for those companies that include them in their surveillance programs.

### RPAS integration in anti-poaching tasks

Considering both the technical and practical aspects we propose three alternatives for RPAS integration into anti-poaching work:

1-As a secret tool for surveillance. Security companies and public entities could use RPAS as a “hidden” tool to monitor systematically poaching hot spots or sensitive areas in order to get data, detect intruders, check rhinoceros presence and safety, as well as provide evidence that could be used on court against poachers. In this case, RPAS must be as discrete as possible. This would entail minimize the noise and camouflage the plane itself and to prevent locals to know about its use.

2-As a supporting tool during poaching incidents. The role of RPAS could be to support ground patrols during the pursuit of poachers, providing real time information about suspect numbers, locations and movements. Images taken may be used as evidence in court if needed. RPAS require less logistics than conventional aircraft, but they still do require some. For this type of very immediate use, technical efforts should be concentrated on developing mobile units integrated in small trailers or 4x4 vehicles that could permit a fast deployment.

3-As a deterrent tool. Security company managers suggested that by making widely known that the area is under constant vigilance by RPAS, it would discourage locals to poach. That would include performing demonstrations to the local communities and appearing in media with awareness campaigns, which could make them afraid and aware that they can be detected even without notice. In this case, it would be convenient to focus the effort with RPAS on farm perimeters surveillance and to get proof of irregular use of the area, giving media coverage to them.

The three alternatives may be combined in different times or areas to optimize the use of the system. For example - keep RPAS use secret until they contribute to catch a poacher and then publicize it widely in the local area.

There is also a fourth use for RPAS, not related to poaching but also involving rhinoceros conservation. RPAS can provide quasi-real time information of habitat changes affecting species movement behavior [9]. Thus, combining high-resolution images of the areas with individuals’ locations, RPAS can contribute to answer ecological questions that have been identified as key conservation factors, such as population density, nutrition and diet [2].

We also foresee a promising field of work using other sensors (like static surveillance cameras and movement detectors) that could work together with RPAS forming an heterogeneous cooperating objects network for sensitive areas surveillance.

### Conclusions-management implications

Our study is the first approach using remotely piloted aircraft systems for anti-poaching tasks and it can be expanded to other areas or species that suffer from the same problem. Some other African and Asiatic countries have rhinoceros poaching problems too, [7], [27] and large mammals such as elephants also suffer from illegal hunting [28]. We have demonstrated that current low cost RPAS present enough technical capabilities to provide useful data, but there are also important practical and technical limitations that must be considered, evaluated and solved by users and authorities before these systems can be deployed in a realistic way (see Table 3 for a summary of the best and worst scenarios). The role RPAS can play in anti-poaching should not be overestimated and investment in this technology should be proportional to the results obtained because the resources for rhinoceros conservation are limited.

**Table 3:** Best and worst scenarios for the use of RPAS in rhinoceros anti-poaching.

Characteristics	Best scenario	Worst scenario
Flight altitude	< 100 m	> 100 m
Range for low-cost RPAS	< 15 km	>15 km
Time period for visual camera	Morning-midday	Evening
Time period for thermal camera	Morning-night	Midday-evening
Meteorology	Wind < 15 km/h	Wind > 15 km/h
	No rain	Rain
	Dry areas	Areas with high humidity
Habitat Characteristics	Open habitats	Thick forest
	Non populated areas	Populated areas
	Low altitude areas	High altitude areas

## Supporting Information

Video S1 Fence surveillance HD video.
(MPG)

Video S2 Thermal camera video.
(MPG)
